# Supplementary material for: Personality-specific carry-over effects on breeding
Source: Proc Biol Sci. 2020 Dec 9;287(1940):20202381. doi: 10.1098/rspb.2020.2381 (PMC7739942; doi:10.1098/rspb.2020.2381)
Supplement: Appendices A-F [file rspb20202381supp1.docx]

Supplementary Material to:

**Personality-specific carry-over effects on breeding**

**Authors:** Stephanie M. Harris; Sébastien Descamps; Lynne U. Sneddon; Milena Cairo; Philip Bertrand; Samantha C. Patrick

Appendix A – Sexing methods

Appendix B – Relationship between boldness and non-breeding activity

Appendix C – Relationship between colony arrival date and lay date

Appendix D – Summary of best supported carry-over effect models, with coefficients of variation

Appendix E – Full carry-over effect model tables

Appendix F – Carry-over effect models run on data subset

*Proceedings of the Royal Society B: Biological Sciences*

DOI: 10.1098/rspb.2020-2381

**Appendix A – Sexing methods**

Blood and feather samples were collected for sex determination upon first capture for each individual. Blood was extracted from the brachial vein using a 26-gauge needle and a heparinized syringe and stored in heparinized vials. The volume of blood taken varied between 0.25ml and 1.5ml because blood was also collected for use in other studies. Several drops of blood were later added to vials containing 0.5ml of 90% ethanol for storage until sexing analysis took place. If blood samples were not otherwise required for other studies, we instead took feather samples, plucking 3-5 body feathers and storing these in sealed plastic bags.

Sex was determined after DNA extraction and polymerase chain reaction (PCR). Genomic DNA was extracted from blood and feathers using DNeasy 96 Blood and Tissue Kit (Qiagen, Hilden, Germany) following the manufacturer’s protocol. Sex was determined using the primers M5 [1] and P8 [2]. These primers amplify the sex-linked CHD-W and CHD-Z genes, which differ in length and result in a single band for males and two bands for females. The M5 primer was 6FAM fluoro-labelled. Polymerase chain reaction (PCR) was performed with Qiagen’s Multiplex PCR Kit following the manufacturer’s protocol, but using 8.4 µL reaction volume. PCR products were mixed with GeneScan 500 LIZ (Applied Biosystems) size standard and Hi-Di formamide. Alleles were separated using capillary electrophoresis on an ABI 3500xl Genetic Analyzer and sizes assigned using GeneMapper software (Applied Biosystems).

**Appendix B – The relationship between boldness and non-breeding activity**

**Table B1.** Summary of models explaining variation in the non-breeding activity (time spent foraging, time in flight) in black-legged kittiwakes. Predictors include boldness, sex, the interval between winter behaviour and boldness measure, and the two-way interactions between boldness and sex, and boldness and interval. Predictors retained in supported models are indicated by estimates for continuous variables or X for categorical variables. Bird ID and season were fitted as random effects in all models. Best supported models, indicated in grey, were those retained when ∆AICc < 2 and where there was no simpler outranking model (the “nesting rule”; [3]).

| **Response** | **Intercept** | **Boldness** | **Sex** | **Boldness x sex** | **Interval** | **Boldness x interval** | **∆AICc** |
| --- | --- | --- | --- | --- | --- | --- | --- |
| Proportion time spent foraging | 0.074 |  |  |  |  |  | 0.00 |
|  | 0.069 |  | X |  |  |  | 2.89 |
|  | -0.116 |  |  |  | 0.097 |  | 4.10 |
|  | 0.073 | 0.032 |  |  |  |  | 4.31 |
|  | -0.129 |  | X |  | 0.098 |  | 7.05 |
|  | 0.058 | 0.035 | X |  |  |  | 7.19 |
|  | -0.121 | -0.042 |  |  | 0.099 |  | 8.44 |
|  | 0.025 | 0.137 | X | X |  |  | 9.63 |
|  | -0.147 | -0.046 | X |  | 0.010 |  | 11.38 |
|  | -0.148 | -0.198 |  |  | 0.104 | 0.010 | 12.20 |
|  | -0.169 | -0.138 | X | X | 0.095 |  | 14.00 |
|  | -0.144 | -0.197 | X |  | 0.104 | 0.010 | 15.26 |
|  | -0.159 | -0.254 | X | X | 0.100 | 0.094 | 18.20 |
| Proportion time in flight | 0.054 |  |  |  |  |  | 0.00 |
|  | 0.029 | 0.271 |  |  |  |  | 0.32 |
|  | 0.286 |  | X |  |  |  | 0.74 |
|  | -0.436 |  |  |  | 0.227 |  | 0.88 |
|  | -0.468 | 0.287 |  |  | 0.230 |  | 1.00 |
|  | -0.200 |  | X |  | 0.222 |  | 1.95 |
|  | 0.209 | 0.242 | X |  |  |  | 2.03 |
|  | -0.291 | 0.260 | X |  | 0.226 |  | 3.00 |
|  | 0.229 | 0.191 | X | X |  |  | 5.08 |
|  | -0.278 | 0.203 | X | X | 0.230 |  | 6.03 |
|  | -0.458 | 0.240 |  |  | 0.226 | 0.031 | 7.05 |
|  | -0.290 | 0.223 | X |  | 0.223 | 0.025 | 9.21 |
|  | -0.279 | 0.179 | X | X | 0.227 | 0.021 | 12.34 |

**Appendix C – Relationship between colony arrival date and lay date**


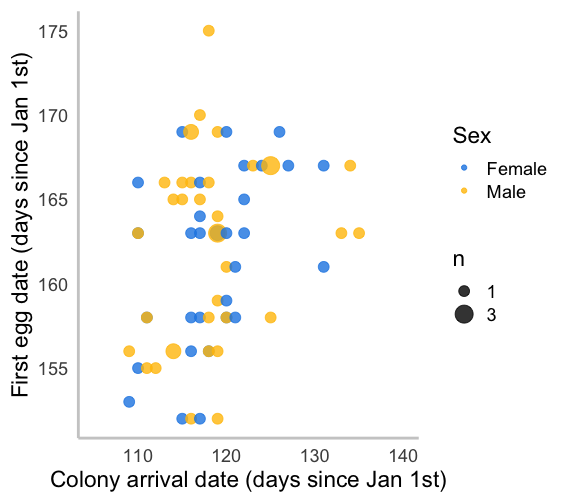
Colony arrival date and first egg date were positively correlated, although not strongly (R = 0.33, p = 0.01; Figure C1). The correlation was stronger among females (R = 0.43, p = 0.01) than among males (R = 0.25, p = 0.12).

**Figure C1.** The correlation between the date on which birds arrived back at the colony in spring and the first date on which their first egg was laid. Point size corresponds to number of individuals.

**Appendix D – Summary of best supported carry-over effect models, with coefficients of variation**

**Table D1**. Summaries of best supported models of carry-over effects of non-breeding activity on colony arrival date, lay date, and offspring survival. Estimates are presented for predictors retained in best supported models only, which were those retained when ∆AICc < 2 and where there was no simpler outranking model (the “nesting rule” [3]). R^2^_m_: marginal coefficient of determination (variance explained by fixed effects); R^2^_c_: conditional coefficient of determination (variance explained by both fixed and random effects). Models were run separately by sex (M: males; F: females). Bird ID and season were fitted as random effects in all models. Arrival date and lay date were controlled for in offspring survival models, and arrival date was controlled for in lay date models (these variables are in grey for their own respective models, where they were not fitted as fixed effects). Full model tables are presented in Appendix E.

| **Response** | **Sex** | **Boldness** | **Foraging** | **Flight** | **Boldness x**  **foraging** | **Boldness x flight** | **Arrival date** | **Lay date** | **∆AICc** | **R^2^_m_** | **R^2^_c_** |
| --- | --- | --- | --- | --- | --- | --- | --- | --- | --- | --- | --- |
| Arrival date | M | 0.28 | 2.40 | 0.01 | -2.15 | -2.06 |  |  | 0.00 | 0.15 | 0.72 |
|  |  |  | 1.82 |  |  |  |  |  | 0.13 | 0.08 | 0.52 |
|  | F | 1.97 |  | 2.48 |  |  |  |  | 0.00 | 0.22 | 0.59 |
| Lay date | M |  | 2.21 | 1.40 |  |  |  |  | 0.00 | 0.13 | 0.27 |
|  |  |  | 1.54 |  |  |  |  |  | 1.13 | 0.08 | 0.26 |
|  | F | 2.76 |  | 2.96 |  | -1.77 |  |  | 0.00 | 0.36 | 0.61 |
| Offspring survival | M | -0.34 | -2.06 |  | 2.23 |  |  | -1.35 | 0.00 | 0.12 | 0.48 |
|  |  | -0.39 | -2.43 |  | 2.01 |  |  |  | 0.25 | 0.09 | 0.48 |
|  |  |  | -1.42 | 1.27 |  |  |  | -1.23 | 1.00 | 0.08 | 0.46 |
|  |  |  | -1.89 | 1.00 |  |  |  |  | 1.27 | 0.07 | 0.47 |
|  |  |  | -2.09 |  |  |  |  | -0.95 | 1.34 | 0.07 | 0.44 |
|  |  |  | -2.35 |  |  |  |  |  | 1.45 | 0.06 | 0.45 |
|  |  |  |  | 2.00 |  |  |  | -1.74 | 1.55 | 0.07 | 0.45 |
|  | F | -1.63 | 1.46 |  |  |  |  |  | 0.00 | 0.05 | 0.68 |
|  |  | -1.48 |  |  |  |  |  | -1.15 | 0.36 | 0.05 | 0.60 |
|  |  | -2.11 |  | -1.02 |  |  |  |  | 0.49 | 0.04 | 0.64 |
|  |  | -1.83 |  |  |  |  |  |  | 0.49 | 0.04 | 0.63 |
|  |  |  | 1.36 |  |  |  |  | -1.27 | 0.68 | 0.04 | 0.66 |
|  |  |  | 1.73 |  |  |  |  |  | 0.85 | 0.03 | 0.70 |
|  |  |  |  |  |  |  |  | -1.71 | 0.95 | 0.03 | 0.59 |
|  |  |  |  |  |  |  | -1.10 |  | 1.83 | 0.01 | 0.60 |

**Appendix E – Full carry-over effect model tables**

**Table E1.** Full model outputs for models examining carry-over effects of non-breeding activity (proportion of time spent foraging, and in flight) on colony arrival date (days since January 1^st^). The top model set is highlighted in grey (those where ∆AIC_C_ < 2 and retained after applying the nesting rule). Models were run separately by sex (M: males; F: females).

| **Response** | **Sex** | **Intercept** | **Boldness** | **Foraging** | **Flight** | **Boldness x**  **foraging** | **Boldness x flight** | **∆AICc** |
| --- | --- | --- | --- | --- | --- | --- | --- | --- |
| Arrival date | M | 118.00 | 0.28 | 2.40 | 0.01 | -2.15 | -2.06 | 0.00 |
|  |  | 118.80 | - | 1.82 | - | - | - | 0.13 |
|  |  | 118.70 | 0.16 | 2.02 | - | -1.27 | - | 0.82 |
|  |  | 118.80 | - | 1.81 | -0.04 | - | - | 1.17 |
|  |  | 118.80 | -0.07 | 1.84 | - | - | - | 1.41 |
|  |  | 118.70 | 0.20 | 2.07 | 0.13 | -1.282 | - | 2.00 |
|  |  | 118.80 | -0.10 | 1.80 | -0.09 | - | - | 2.43 |
|  |  | 118.40 | -0.14 | 1.90 | -0.20 | - | -0.94 | 2.92 |
|  |  | 118.90 | - | - | -0.90 | - | - | 3.87 |
|  |  | 118.90 | - | - | - | - | - | 3.88 |
|  |  | 118.90 | -0.44 | - | -1.05 | - | - | 4.73 |
|  |  | 118.90 | -0.08 | - | - | - | - | 4.91 |
|  |  | 118.70 | -0.46 | - | -1.17 | - | -0.73 | 5.33 |
|  | F | 119.20 | 1.97 | - | 2.48 | - | - | 0.00 |
|  |  | 119.10 | 1.92 | -0.23 | 2.43 | - | - | 1.59 |
|  |  | 119.10 | 1.97 | - | 2.50 | - | -0.02 | 1.63 |
|  |  | 119.00 | - | - | 2.08 | - | - | 2.78 |
|  |  | 119.10 | 1.92 | -0.23 | 2.43 | - | -0.01 | 3.40 |
|  |  | 119.20 | 1.95 | -0.21 | 2.48 | 0.20 | - | 3.49 |
|  |  | 118.90 | - | -0.53 | 1.99 | - | - | 3.81 |
|  |  | 119.20 | 1.94 | -0.22 | 2.45 | 0.23 | 0.08 | 5.43 |
|  |  | 119.60 | 1.41 | - | - | - | - | 5.52 |
|  |  | 119.50 | 1.26 | -0.64 | - | - | - | 6.31 |
|  |  | 119.40 | - | - | - | - | - | 6.48 |
|  |  | 119.30 | - | -0.80 | - | - | - | 6.78 |
|  |  | 119.50 | 1.23 | -0.67 | - | -0.22 | - | 7.93 |

**Table E2.** Full model outputs for models examining carry-over effects of non-breeding activity (proportion of time spent foraging, and in flight) on lay date (days since January 1^st^). The top model set is highlighted in grey (those where ∆AIC_C_ < 2 and retained after applying the nesting rule). Models were run separately by sex (M: males; F: females).

| **Response** | **Sex** | **Intercept** | **Boldness** | **Foraging** | **Flight** | **Boldness x**  **foraging** | **Boldness x flight** | **Arrival date** | **∆AICc** |
| --- | --- | --- | --- | --- | --- | --- | --- | --- | --- |
| Lay date | M | 162.94 | - | 2.21 | 1.40 | - | - | - | 0.00 |
|  |  | 162.89 | 0.96 | 2.43 | 1.85 | - | - | - | 0.21 |
|  |  | 162.89 | - | 1.98 | 1.41 | - | - | 0.86 | 0.46 |
|  |  | 162.85 | 0.97 | 2.20 | 1.86 | - | - | 0.87 | 0.85 |
|  |  | 162.61 | 0.88 | 2.47 | 1.72 | - | -0.82 | - | 0.90 |
|  |  | 162.96 | - | 1.54 | - | - | - | - | 1.13 |
|  |  | 162.92 | - | 1.31 | - | - | - | 0.85 | 1.42 |
|  |  | 162.91 | 0.84 | 2.35 | 1.72 | 0.49 | - | - | 1.48 |
|  |  | 162.56 | 0.90 | 2.25 | 1.74 | - | -0.78 | 0.84 | 1.82 |
|  |  | 162.86 | 0.79 | 2.03 | 1.67 | 0.72 | - | 1.04 | 1.97 |
|  |  | 162.95 | 0.32 | 1.55 | - | - | - | - | 2.35 |
|  |  | 162.64 | 0.85 | 2.44 | 1.70 | 0.16 | -0.73 | - | 2.38 |
|  |  | 162.98 | - | - | - | - | - | 1.29 | 2.47 |
|  |  | 162.90 | 0.32 | 1.32 | - | - | - | 0.85 | 2.80 |
|  |  | 162.97 | 0.17 | 1.51 | - | 0.84 | - | - | 2.84 |
|  |  | 162.92 | 0.15 | 1.20 | - | 1.07 | - | 1.10 | 2.98 |
|  |  | 162.66 | 0.80 | 2.13 | 1.66 | 0.46 | -0.53 | 0.96 | 3.29 |
|  |  | 162.98 | - | - | 0.51 | - | - | 1.37 | 3.41 |
|  |  | 163.04 | - | - | - | - | - | - | 3.49 |
|  |  | 162.96 | 0.31 | - | - | - | - | 1.30 | 3.66 |
|  |  | 162.96 | 0.55 | - | 0.71 | - | - | 1.41 | 4.39 |
|  |  | 163.04 | - | - | 0.32 | - | - | - | 4.47 |
|  |  | 163.03 | 0.29 | - | - | - | - | - | 4.51 |
|  |  | 162.72 | 0.49 | - | 0.60 | - | -0.62 | 1.39 | 5.31 |
|  |  | 163.02 | 0.45 | - | 0.48 | - | - | - | 5.38 |
|  |  | 162.78 | 0.38 | - | 0.36 | - | -0.68 | - | 6.01 |
|  | F | 161.63 | 2.76 | - | 2.96 | - | -1.77 | - | 0.00 |
|  |  | 161.59 | 2.58 | - | 2.81 | - | -1.76 | 0.49 | 1.85 |
|  |  | 161.63 | 2.72 | -0.24 | 2.90 | - | -1.75 | - | 2.51 |
|  |  | 161.59 | 2.54 | -0.24 | 2.74 | - | -1.74 | 0.50 | 4.66 |
|  |  | 162.06 | 2.44 | - | 1.86 | - | - | - | 4.78 |
|  |  | 161.65 | 2.73 | -0.25 | 2.90 | 0.26 | -1.65 | - | 5.07 |
|  |  | 162.02 | 2.24 | - | 1.69 | - | - | 0.58 | 6.09 |
|  |  | 162.07 | 2.38 | -0.34 | 1.78 | - | - | - | 6.69 |
|  |  | 162.05 | 2.54 | -0.34 | 2.04 | 1.04 | - | - | 7.25 |
|  |  | 161.61 | 2.55 | -0.25 | 2.74 | 0.29 | -1.64 | 0.52 | 7.53 |
|  |  | 162.04 | 1.57 | - | - | - | - | 1.23 | 8.21 |
|  |  | 162.03 | 2.18 | -0.33 | 1.62 | - | - | 0.57 | 8.27 |
|  |  | 162.14 | 1.90 | - | - | - | - | - | 8.52 |
|  |  | 162.01 | 2.33 | -0.32 | 1.86 | 1.04 | - | 0.60 | 9.13 |
|  |  | 162.15 | 1.83 | -0.68 | - | - | - | - | 9.50 |
|  |  | 162.06 | 1.53 | -0.60 | - | - | - | 1.15 | 9.64 |
|  |  | 161.96 | - | - | - | - | - | 1.85 | 10.50 |
|  |  | 162.15 | 1.87 | -0.70 | - | 0.58 | - | - | 10.92 |
|  |  | 162.05 | 1.56 | -0.62 | - | 0.70 | - | 1.24 | 11.10 |
|  |  | 161.93 | - | - | 0.76 | - | - | 1.68 | 11.27 |
|  |  | 162.00 | - | -0.68 | - | - | - | 1.74 | 11.47 |
|  |  | 161.96 | - | -0.58 | 0.66 | - | - | 1.61 | 12.69 |
|  |  | 162.03 | - | - | 1.30 | - | - | - | 12.74 |
|  |  | 162.13 | - | - | - | - | - | - | 13.31 |
|  |  | 162.14 | - | -0.86 | - | - | - | - | 13.61 |
|  |  | 162.06 | - | -0.68 | 1.15 | - | - | - | 13.68 |

**Table E3.** Full model outputs for models examining carry-over effects of non-breeding activity (proportion of time spent foraging, and in flight) on offspring survival (days since hatching). The top model set is highlighted in grey (those where ∆AIC_C_ < 2 and retained after applying the nesting rule). Models were run separately by sex (M: males; F: females). Table continues onto the next page with results for females.

| **Response** | **Sex** | **Intercept** | **Boldness** | **Foraging** | **Flight** | **Boldness x**  **foraging** | **Boldness x flight** | **Arrival date** | **Lay date** | **∆AICc** |
| --- | --- | --- | --- | --- | --- | --- | --- | --- | --- | --- |
| Offspring survival | M | 13.12 | -0.34 | -2.06 | - | 2.23 | - | - | -1.35 | 0.00 |
|  |  | 13.16 | 0.11 | -1.46 | 1.12 | 2.06 | - | - | -1.60 | 0.16 |
|  |  | 12.94 | -0.39 | -2.43 | - | 2.01 | - | - | - | 0.25 |
|  |  | 12.95 | -0.13 | -2.11 | 0.67 | 1.88 | - | - | - | 0.57 |
|  |  | 13.43 | 0.08 | -1.59 | 1.12 | 2.38 | 0.76 | - | -1.53 | 0.75 |
|  |  | 13.12 | -0.34 | -2.14 | - | 2.29 | - | 0.29 | -1.40 | 0.77 |
|  |  | 13.31 | -0.16 | -2.24 | 0.70 | 2.30 | 0.97 | - | - | 0.79 |
|  |  | 12.94 | -0.39 | -2.44 | - | 2.01 | - | 0.03 | - | 0.86 |
|  |  | 13.08 | - | -1.42 | 1.27 | - | - | - | -1.23 | 1.00 |
|  |  | 13.16 | 0.11 | -1.53 | 1.12 | 2.12 | - | 0.30 | -1.65 | 1.15 |
|  |  | 12.92 | - | -1.89 | 1.00 | - | - | - | - | 1.27 |
|  |  | 13.02 | - | -2.09 | - | - | - | - | -0.95 | 1.34 |
|  |  | 12.95 | -0.13 | -2.11 | 0.67 | 1.88 | - | 0.01 | - | 1.38 |
|  |  | 12.90 | - | -2.35 | - | - | - | - | - | 1.45 |
|  |  | 13.12 | - | - | 2.00 | - | - | - | -1.74 | 1.55 |
|  |  | 13.09 | 0.63 | -1.22 | 1.58 | - | - | - | -1.36 | 1.57 |
|  |  | 13.09 | - | -1.36 | 1.26 | - | - | -0.26 | -1.20 | 1.57 |
|  |  | 12.93 | - | -1.76 | 0.99 | - | - | -0.42 | - | 1.62 |
|  |  | 12.91 | - | -2.22 | - | - | - | -0.43 | - | 1.62 |
|  |  | 13.11 | 0.94 | - | 2.31 | - | - | - | -1.83 | 1.69 |
|  |  | 13.03 | - | -2.00 | - | - | - | -0.30 | -0.92 | 1.71 |
|  |  | 13.33 | -0.17 | -2.34 | 0.69 | 2.39 | 1.05 | 0.27 | - | 1.71 |
|  |  | 12.91 | 0.38 | -1.80 | 1.17 | - | - | - | - | 1.82 |
|  |  | 13.48 | 0.07 | -1.75 | 1.13 | 2.54 | 0.91 | 0.51 | -1.60 | 1.85 |
|  |  | 13.12 | - | - | 1.90 | - | - | -0.59 | -1.61 | 1.85 |
|  |  | 12.89 | 0.88 | - | 2.20 | - | -0.65 | - | -1.89 | 2.06 |
|  |  | 12.90 | -0.03 | -2.35 | - | - | - | - | - | 2.09 |
|  |  | 13.02 | 0.05 | -2.08 | - | - | - | - | -0.96 | 2.14 |
|  |  | 12.92 | 0.60 | -1.15 | 1.53 | - | -0.50 | - | -1.44 | 2.21 |
|  |  | 13.12 | 0.89 | - | 2.22 | - | - | -0.47 | -1.72 | 2.23 |
|  |  | 12.93 | 0.37 | -1.68 | 1.16 | - | - | -0.41 | - | 2.34 |
|  |  | 13.09 | 0.62 | -1.17 | 1.57 | - | - | -0.23 | -1.33 | 2.34 |
|  |  | 12.82 | 0.36 | -1.78 | 1.13 | - | -0.26 | - | - | 2.36 |
|  |  | 12.92 | -0.03 | -2.22 | - | - | - | -0.43 | - | 2.42 |
|  |  | 13.03 | 0.04 | -2.00 | - | - | - | -0.30 | -0.92 | 2.68 |
|  |  | 12.91 | - | - | 1.78 | - | - | -0.98 | - | 2.72 |
|  |  | 12.88 | 0.82 | - | 2.09 | - | -0.69 | -0.52 | -1.78 | 2.77 |
|  |  | 12.86 | - | - | 1.93 | - | - | - | - | 2.90 |
|  |  | 12.85 | 0.78 | - | 2.19 | - | - | - | - | 3.01 |
|  |  | 12.81 | 0.34 | -1.64 | 1.11 | - | -0.33 | -0.46 | - | 3.04 |
|  |  | 12.89 | 0.70 | - | 2.03 | - | - | -0.91 | - | 3.07 |
|  |  | 12.91 | 0.59 | -1.09 | 1.52 | - | -0.53 | -0.28 | -1.40 | 3.18 |
|  |  | 12.71 | 0.74 | - | 2.12 | - | -0.41 | - | - | 3.29 |
|  |  | 13.04 | - | - | - | - | - | -0.97 | -1.45 | 3.44 |
|  |  | 12.71 | 0.64 | - | 1.92 | - | -0.51 | -0.96 | - | 3.48 |
|  |  | 13.03 | - | - | - | - | - | - | -1.66 | 3.62 |
|  |  | 13.04 | 0.13 | - | - | - | - | -0.95 | -1.47 | 4.15 |
|  |  | 13.03 | 0.18 | - | - | - | - | - | -1.68 | 4.16 |

| **Response** | **Sex** | **Intercept** | **Boldness** | **Foraging** | **Flight** | **Boldness x**  **foraging** | **Boldness x flight** | **Arrival date** | **Lay date** | **∆AICc** |
| --- | --- | --- | --- | --- | --- | --- | --- | --- | --- | --- |
| Offspring survival | F | 14.41 | -1.63 | 1.46 | - | - | - | - | - | 0.00 |
|  |  | 14.57 | -1.48 | - | - | - | - | - | -1.15 | 0.36 |
|  |  | 14.46 | -1.85 | 1.36 | -0.79 | - | - | - | - | 0.43 |
|  |  | 14.53 | -1.42 | 1.27 | - | - | - | - | -0.80 | 0.44 |
|  |  | 14.46 | -2.11 | - | -1.02 | - | - | - | - | 0.49 |
|  |  | 14.40 | -1.83 | - | - | - | - | - | - | 0.49 |
|  |  | 14.43 | -1.57 | 1.42 | - | - | - | -0.27 | - | 0.66 |
|  |  | 14.65 | - | 1.36 | - | - | - | - | -1.27 | 0.68 |
|  |  | 14.57 | -1.76 | - | -0.70 | - | - | - | -0.87 | 0.77 |
|  |  | 14.45 | -1.71 | - | - | - | - | -0.50 | - | 0.81 |
|  |  | 14.49 | - | 1.73 | - | - | - | - | - | 0.85 |
|  |  | 14.72 | -2.49 | - | -1.55 | - | 0.93 | - | - | 0.91 |
|  |  | 14.40 | -1.64 | 1.45 | - | -0.12 | - | - | - | 0.92 |
|  |  | 14.60 | -1.41 | - | - | - | - | -0.34 | -1.11 | 0.93 |
|  |  | 14.69 | - | - | - | - | - | - | -1.71 | 0.95 |
|  |  | 14.47 | -2.09 | - | -1.01 | - | - | -0.04 | - | 1.02 |
|  |  | 14.73 | - | - | - | - | - | -0.75 | -1.59 | 1.14 |
|  |  | 14.54 | - | 1.61 | - | - | - | -0.72 | - | 1.15 |
|  |  | 14.53 | -1.65 | 1.24 | -0.60 | - | - | - | -0.58 | 1.17 |
|  |  | 14.68 | - | 1.29 | - | - | - | -0.56 | -1.21 | 1.23 |
|  |  | 14.46 | -1.88 | 1.35 | -0.82 | - | - | 0.08 | - | 1.26 |
|  |  | 14.66 | -2.17 | 1.23 | -1.23 | - | 0.72 | - | - | 1.31 |
|  |  | 14.54 | -1.38 | 1.25 | - | - | - | -0.20 | -0.79 | 1.34 |
|  |  | 14.51 | - | 1.73 | -0.21 | - | - | - | - | 1.36 |
|  |  | 14.64 | - | 1.38 | 0.09 | - | - | - | -1.27 | 1.42 |
|  |  | 14.72 | -2.25 | - | -1.30 | - | 0.75 | - | -0.42 | 1.42 |
|  |  | 14.68 | - | - | 0.05 | - | - | - | -1.70 | 1.48 |
|  |  | 14.58 | -1.74 | - | -0.68 | - | - | -0.06 | -0.88 | 1.55 |
|  |  | 14.46 | -1.86 | 1.34 | -0.81 | -0.17 | - | - | - | 1.60 |
|  |  | 14.53 | -1.42 | 1.25 | - | -0.04 | - | - | -0.81 | 1.60 |
|  |  | 14.72 | -2.48 | - | -1.54 | - | 0.92 | -0.02 | - | 1.71 |
|  |  | 14.43 | -1.57 | 1.41 | - | -0.15 | - | -0.31 | - | 1.81 |
|  |  | 14.55 | - | - | - | - | - | -1.10 | - | 1.83 |
|  |  | 14.54 | - | 1.63 | -0.04 | - | - | -0.66 | - | 1.88 |
|  |  | 14.72 | - | - | 0.19 | - | - | -0.74 | -1.61 | 1.88 |
|  |  | 14.46 | - | - | - | - | - | - | - | 2.05 |
|  |  | 14.66 | - | 1.32 | 0.21 | - | - | -0.56 | -1.24 | 2.20 |
|  |  | 14.66 | -2.06 | 1.19 | -1.10 | - | 0.63 | - | -0.22 | 2.20 |
|  |  | 14.53 | -1.66 | 1.23 | -0.61 | - | - | 0.05 | -0.59 | 2.28 |
|  |  | 14.49 | - | - | -0.38 | - | - | - | - | 2.29 |
|  |  | 14.55 | - | - | -0.13 | - | - | -1.00 | - | 2.33 |
|  |  | 14.65 | -2.21 | 1.22 | -1.26 | - | 0.72 | 0.10 | - | 2.44 |
|  |  | 14.73 | -2.22 | - | -1.27 | - | 0.74 | -0.05 | -0.43 | 2.50 |
|  |  | 14.70 | -2.23 | 1.18 | -1.28 | 0.22 | 0.83 | - | - | 2.52 |
|  |  | 14.53 | -1.65 | 1.23 | -0.60 | -0.10 | - | - | -0.58 | 2.61 |
|  |  | 14.45 | -1.88 | 1.33 | -0.82 | -0.17 | - | 0.04 | - | 2.71 |
|  |  | 14.55 | -1.38 | 1.22 | - | -0.06 | - | -0.23 | -0.80 | 2.77 |
|  |  | 14.66 | -2.07 | 1.18 | -1.11 | - | 0.62 | 0.06 | -0.23 | 3.66 |

(Table E3 continued):

**Appendix F – Carry-over effect models run on data subset**

The interval between collection of personality assays and non-breeding activity data ranged from 0 to 5 years. We therefore reran carry-over effects models with a conservative subset of the data, where the interval between measurements was two years or less. For each analysis (e.g. for effects of colony arrival date in males, then in females, etc.) we fitted the top-ranking models (those presented in supplementary Table D1) on the subset of data. We compared the parameter estimates from these models to the estimates presented in Table 2 of the main paper, to see whether results differed substantially in strength and direction. We present both sets of estimates ± standard error for all variables in initial models in Table F1 below. Parameter estimates from the subset data matched those from the full dataset well in terms of both direction and strength: there are some exceptions, for example the effect of boldness on offspring survival of males is stronger when we subset the data, although the direction of the relationship remained the same. We therefore interpret this as strong evidence that our findings are not driven by the intervals between boldness tests and non-breeding tracking in our dataset.

**Table F1.** Estimates and standard errors for all retained variables, as run on a conservative subset of data where the interval between boldness and winter activity measurements was 2 years of less.

|  |  | **Males** | | **Females** | |
| --- | --- | --- | --- | --- | --- |
| **Response** | **Predictor** | **Full data** | **Subset data** | **Full data** | **Subset data** |
| Colony arrival date | Intercept | 118.41 ± 2.30 | 117.29 ± 1.77 | 119.16 ± 1.33 | 117.95 ± 1.70 |
|  | Boldness | 0.28 ± 0.99 | 0.17 ± 1.31 | 1.97 ± 0.94 | 2.28 ± 1.01 |
|  | Foraging | 2.12 ± 0.95 | 2.15 ± 1.09 |  |  |
|  | Flight | 0.00 ± 1.04 | -0.49 ± 1.50 | 2.48 ± 0.88 | 1.76 ± 1.08 |
|  | Boldness x foraging | -2.15 ± 1.03 | -1.97 ± 1.40 |  |  |
|  | Boldness x flight | -2.06 ± 1.10 | -2.00 ± 1.67 |  |  |
| Lay date | Intercept | 162.94 ± 1.33 | 160.89 ± 1.17 | 161.63 ± 1.33 | 160.65 ± 1.78 |
|  | Boldness |  |  | 2.76 ± 0.63 | 2.36 ± 0.94 |
|  | Foraging | 1.97 ± 0.95 | 1.76 ± 1.69 |  |  |
|  | Flight | 1.40 ± 0.95 | 0.15 ± 1.43 | 2.96 ± 0.79 | 2.45 ± 1.40 |
|  | Boldness x foraging |  |  |  |  |
|  | Boldness x flight |  |  | -1.77 ± 0.62 | -1.46 ± 1.13 |
|  | Arrival date |  |  |  |  |
| Offspring survival | Intercept | 13.02 ± 2.95 | 13.53 ± 5.10 | 14.52 ± 3.62 | 14.51 ± 5.44 |
|  | Boldness | -0.36 ± 1.23 | -1.86 ± 1.78 | -1.75 ± 1.37 | -1.88 ± 1.77 |
|  | Foraging | -2.06 ± 1.32 | -2.15 ± 1.74 | 1.50 ± 1.32 | 2.38 ± 1.80 |
|  | Flight | 1.39 ± 1.44 | 1.83 ± 2.01 | -1.02 ± 1.50 | -0.54 ± 2.02 |
|  | Boldness x foraging | 2.13 ± 1.32 | 2.06 ± 1.71 |  |  |
|  | Boldness x flight |  |  |  |  |
|  | Arrival date |  |  | -1.10 ± 1.52 | -1.90 ± 2.09 |
|  | Lay date | -1.31 ± 1.41 | -1.24 ± 1.70 | -1.35 ± 1.50 | -1.26 ± 1.94 |

**References**

1. Bantock TM, Prys-Jones RP, Lee PLM. 2008 New and improved molecular sexing methods for museum bird specimens. *Mol. Ecol. Resour.* **8**, 519–528. (doi:10.1111/j.1471-8286.2007.01999.x)

2. Griffiths R, Double MC, Orr K, Dawson RJG. 1998 A DNA test to sex most birds. *Mol. Ecol.* **7**, 1071–1075. (doi:10.1046/j.1365-294x.1998.00389.x)

3. Arnold, T (2010) Uninformative parameters and model selection using Akaike's Information Criterion. *J. of Wildlife Manag.* **75**, 1175-1178.*,*
